# Defects in the NC2 repressor affect both canonical and non-coding RNA polymerase II transcription initiation in yeast

**DOI:** 10.1186/s12864-016-2536-2

**Published:** 2016-03-03

**Authors:** Natalia Gómez-Navarro, Antonio Jordán-Pla, Francisco Estruch, José E. Pérez-Ortín

**Affiliations:** Departamento de Bioquímica y Biología Molecular, Facultad de Biológicas and ERI Biotecmed, Universitat de València, Dr. Moliner 50, E-46100 Burjassot, Valencia Spain; Present address: MRC Laboratory of Molecular Biology, Francis Crick Avenue, Cambridge, CB2 0QH UK; Present address: Department of Molecular Biosciences, The Wenner-Gren Institute, Stockholm University, Stockholm, SE-106 91 Sweden

**Keywords:** NC2, Transcription initiation, Yeast, TATA, TATA-like, Cryptic transcript

## Abstract

**Background:**

The formation of the pre-initiation complex in eukaryotic genes is a key step in transcription initiation. The TATA-binding protein (TBP) is a universal component of all pre-initiation complexes for all kinds of RNA polymerase II (RNA pol II) genes, including those with a TATA or a TATA-like element, both those that encode proteins and those that transcribe non-coding RNAs. Mot1 and the negative cofactor 2 (NC2) complex are regulators of TBP, and it has been shown that depletion of these factors in yeast leads to defects in the control of transcription initiation that alter cryptic transcription levels in selected yeast loci.

**Results:**

In order to cast light on the molecular functions of NC2, we performed genome-wide studies in conditional mutants in yeast NC2 essential subunits Ydr1 and Bur6. Our analyses show a generally increased level of cryptic transcription in all kinds of genes upon depletion of NC2 subunits, and that each kind of gene (canonical or ncRNAs, TATA or TATA-like) shows some differences in the cryptic transcription pattern for each NC2 mutant.

**Conclusions:**

We conclude that NC2 plays a general role in transcription initiation in RNA polymerase II genes that is related with its known TBP interchange function from free to promoter bound states. Therefore, loss of the NC2 function provokes increases in cryptic transcription throughout the yeast genome. Our results also suggest functional differences between NC2 subunits Ydr1 and Bur6.

## Background

Transcription of eukaryotic genes requires the assembly of a multiprotein preinitiation complex (PIC), including RNA polymerase II (RNA pol II) and general transcription factors (GTFs). All known eukaryotic genes require the recruitment of the TATA-binding protein (TBP) for PIC formation [1]. However, only a minority of RNA pol II genes have a consensus binding site for TBP, the TATA box. For instance, in the yeast *Saccharomyces cerevisiae*, only 15-20 % of the RNA pol II genes that code for proteins have a canonical TATA box [1–3]. Also, non-coding RNA (ncRNA) genes transcribed by RNA pol II can have either TATA or TATA-like promoters [3].

At the TATA-like promoters, TBP arrives as part of the TFIID complex while the SAGA (Spt-Ada-Gcn5-acetyltransferase) complex recruits TBP at the TATA box-containing genes [2, 4]. Binding of TBP to the TATA box is stabilized by TFIIA and leads to the recruitment of TFIIB [5], whereas the TBP-associated factors (TAFs) of TFIID facilitate binding of TBP specifically to TATA-like promoters [6].

Promoter occupancy by TBP is also subjected to negative regulation which is generally associated with inaccessibility due to chromatin structure [7]. Proper integrity of chromatin structure is required for accurate transcription initiation and mutants with disrupted chromatin show spurious transcription initiations from cryptic promoters across the genome, including initiation at intragenic locations [8–11]. Other mechanisms of repression operate through the core promoter and general transcription factor interactions [12]. This type of negative regulation is exemplified by the actions of Mot1 and negative cofactor 2 (NC2). Mot1 is a Snf2 family ATPase that removes or redistributes TBP from promoters. NC2 is a heterodimer of NC2α (Bur6 in yeast) and NC2β (Ydr1 in yeast) [13, 14] that inhibits PIC formation by interfering with the binding of TFIIA and TFIIB with TBP [15–17]. In yeast, a genome-wide analysis has demonstrated the strong co-localization of NC2, Mot1 and TBP at many active promoters. Further, a protein complex has been purified from chromatin extracts that contains NC2, Mot1, TBP and 20–70 bp of DNA [18].

Besides their roles in transcriptional repression, both Mot1 and NC2 are involved in gene activation. The participation of Mot1 and NC2 in gene activation has been related with the displacement of TBP from inappropriate genomic locations (see [19] and references therein). The observation that Mot1 and NC2 selectively target TATA-containing genes, but not TATA-like genes, for negative regulation [2] is in agreement with the requirement of Mot1 to remove TBP from the preferred TATA promoters, which increases the amount of TBP available to bind intrinsically disfavored (TATA-like) sites [20].

Recently Koster et al. found that depletion of Mot1 and NC2 in mutants with disrupted chromatin leads to intragenic transcription in the *FLO8* gene. The effect of Mot1 and NC2 in suppressing intragenic transcription is a characteristic specific to cryptic TATA-containing promoters wherein they remove TBP from sites that have been exposed upon chromatin structure disruption. In contrast, depletion of Mot1 or NC2 causes a decreased expression from intragenic TATA-like promoters, which is consistent with a role of Mot1 and NC2 in the redistribution of TBP from TATA-containing to TATA-like binding sites [21].

The function of Mot1 and NC2 responsible for removing TBP from cryptic sites is also important for restricting antisense ncRNA synthesis. Koster and Timmers [22] have shown that Mot1 and NC2 restrict the formation of PICs from 3′-end of genes by TBP displacement, which limits antisense ncRNA production.

In this study we performed a genome-wide transcriptomic analysis to show that disruption of NC2 function by means of shutting off either of its two protein subunits in yeast provokes an increase in cryptic transcription both around and inside any kind of RNA pol II gene. We conclude that NC2 is necessary for the correct location of the PIC and that its absence induces cryptic (sense and antisense) initiations upstream of canonical promoters, in terminator regions and alongside the transcribed region.

## Results

### Ydr1 depletion provokes increased cryptic transcription in *S. cerevisiae*

With the aim of obtaining a yeast strain that lacks Ydr1 activity, and because it is an essential gene which prevents the use deletion mutants, we constructed a conditional expression version of the *YDR1* gene by replacing its natural promoter with the *GAL10* promoter. In this strain we analyzed the Ydr1 levels by taking advantage of the three copies of the HA epitope that had been added to the N-terminal end of the protein during the substitution of *YDR1* promoter for *GAL10* [23]. After a 4 h incubation in dextrose, we were unable to detect any HA-Ydr1 protein by Western blot [24]. Accordingly, this strain was unable to grow in glucose.

**Fig. 1:**
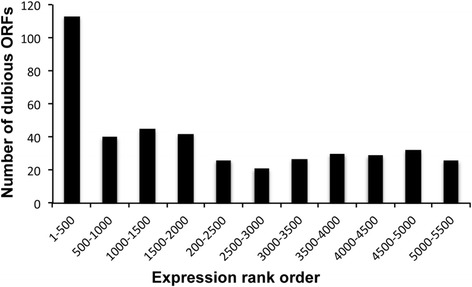
Dubious ORFs are up-regulated under Ydr1 depletion conditions. Histogram of the number ORFs classified as “dubious” by SGD, included in each group of 500 ORF, detected by yeast ORF macroarrays and ranked in decreasing order of expression level in the *ydr1* mutant vs. the wild type

In a first approach to analyze the transcriptomic effects upon depletion of NC2, we used yeast ORF macroarrays [25]. After 4 h in glucose, 509 genes showed at least a two-fold change in their expression levels in the *P*_*GAL10*_-*YDR1* strain compared to the wild-type control. The shutoff of Ydr1 resulted mainly in increased transcript levels (414 genes vs. only 95 genes that exhibited a lower expression), which suggests that NC2 has mostly a negative effect on gene expression [24]. We also noticed that the list of genes with increased transcript levels under Ydr1p depletion conditions contained an abnormally high proportion of genes in the “dubious ORF” category, as stated in the Saccharomyces Genome Database (SGD). As Fig. 1 depicts, from the 431 “dubious” ORFs detected in our analysis, 113 were included among the 500 ORFs with the highest expression fold-change in the mutant compared to the wild-type strain (of a total of 5803 ORFs analyzed in the macroarray experiment).

These “dubious” ORF were, by different criteria, unlikely to encode an expressed protein (according SGD definitions). They are often small and partially overlap with larger (and, likely, expressed) ORFs. It should be noted that the macroarrays used for transcriptome analyses contained probes that covered only the ORF, and thus the full transcripts that exceed ORF limits can overlap proximal probes that cover dubious ORFs. Thus, we suspected that the increase in dubious ORF transcription would more likely reflect the increased cryptic transcription in the flanks of the canonical genes in the *ydr1* mutant.

### High-resolution tiling array analysis shows an increase in different kinds of cryptic transcription upon NC2 disruption

As a second approach, and with the aim of obtaining an expanded and more detailed view of the transcriptional impact of the depletion of NC2 subunits, we applied the same experimental setup by high-density strand-specific tiling arrays. This allowed us to analyze the exact position and expression level of each known or unknown transcript.

**Fig. 2:**
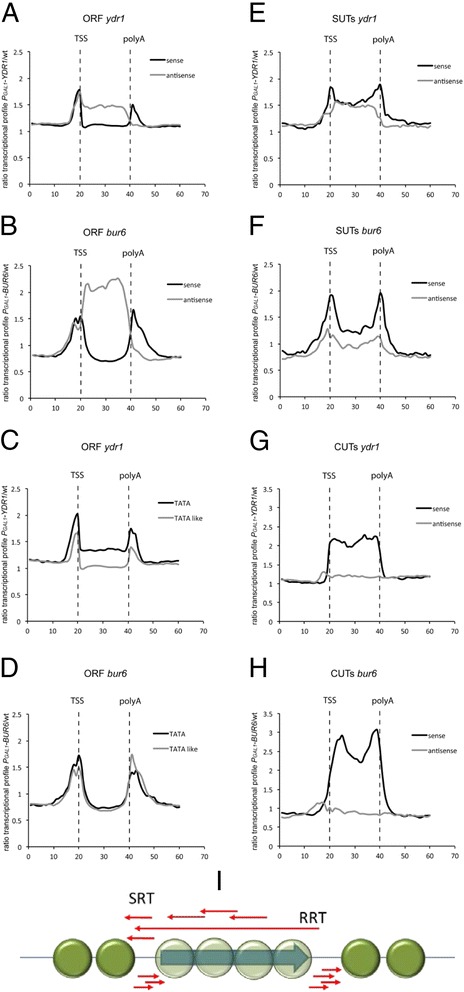
NC2 shutoff provokes an increase in non-coding RNA transcripts. Metagene representation of the average transcription profiles for each analyzed gene class. Ten units correspond to 200 bp. In between TSS (transcription start site) and polyA (polyadenylation site), the entire gene lengths are normalized to 20 units (20–40 on the abscissa scale). The ordinates scale represents the ratio between the mutant and wild-type signals. **a**–**b**) Canonical ORFs show an increase in both the short sense transcripts in the 5′ and 3′ flanks and in antisense transcripts when blocking the transcription of both NC2 subunits. **c**–**d**) Only minor differences are seen between TATA and TATA-like genes when blocking NC2 transcripts. **e**–**h**) Cryptic mapped transcripts (SUTs and CUTs) show similar effects to those observed in canonical genes after shutting down NC2 subunits. **a**, **c**, **e**, **g**: pGAL-YDR1 shutoff; **b**, **d**, **f**, **h**: pGAL-BUR6 shutoff. **i**) Interpretation of the observed results. The cryptic transcripts (red arrows) over a scheme for a canonical yeast gene with its typical nucleosomal organization. Putative SRT (Ssu72-restricted transcripts) and RRT (Rco1-restricted transcripts) are marked. See the main text for a discussion

Figure 2 shows the average metagene profiles for all the canonical yeast genes for which their transcription start sites (TSS) and polyadenylation sites (pA) were mapped (5423 genes). Figure 2a & b (black lines) illustrates how both shutoff mutants presented a higher relative signal in the mutant strain compared to the wild type around the promoter and terminator regions, which indicates the existence of limited regions of sense-oriented cryptic transcription. Apart from this, the absence of an intact NC2 also clearly facilitated antisense transcription inside the coding region, especially for the *bur6* mutant (gray lines). It was also remarkable that in the antisense orientation a signal accumulated in the 5′ flank that was not present at the 3′ flank in either of the two mutants (Fig. 2a & b). The height and shape of the profile at the gene ends in both mutants slightly differed: *bur6* depletion (Fig. 2b) appeared to have a stronger effect than *ydr1* depletion (Fig. 2a) because cryptic transcription entered the coding region and the 3′ peak was relatively more pronounced than the 5′ one.

As yeast genes were organized into two distinct classes according to their promoter organization and different behavior during PIC formation [2], we wondered if the absence of NC2 in cryptic transcription would differently affect both kinds of promoters. Figure 2c depicts how TATA-containing genes (918 genes) exhibited an increase in the signal in the *ydr1* mutant, both at the gene borders and within the transcribed region, whereas TATA-like genes (4060 genes) showed only cryptic transcripts exclusively in the gene borders described before (Fig. 2a). However, in the *bur6* mutant, no major differences were found between both gene types, and there were clear peaks at both gene borders (Fig. 2d).

As RNA pol II also transcribes ncRNAs, we wished to analyze the behavior of the NC2 shutoff mutants in this kind of genes. For this we used previously described lists [26] of stable untranslated transcripts (SUTs, 847 ncRNAs) and cryptic unstable transcripts (CUTs, 925 ncRNAs). Given the small number and low expression level of these ncRNAs, the metagene profiles had a higher background noise than the average protein-coding gene. Nevertheless, it became clear that the effect of NC2 depletion was similar to that in canonical genes. The differences were that the 5′ and 3′ peaks were higher (especially in CUTs), and positioned exactly at the TSS and pA (especially in SUTs), or even inside the transcribed region (in CUTs), and not just upstream as in canonical genes. There was also some antisense transcription in SUTs (Fig. 2e & f), but none in CUTs (Fig. 2g & h).

Overall, our results led us to conclude that lack of NC2 increased fuzziness in the selection of the start site in all RNA pol II-dependent transcription, regardless of their protein-coding potential. This was especially relevant in the nucleosome-depleted regions at the 5′ and 3′ flanks of yeast genes (Fig. 2i), but also within the coding region.

**Fig. 3:**
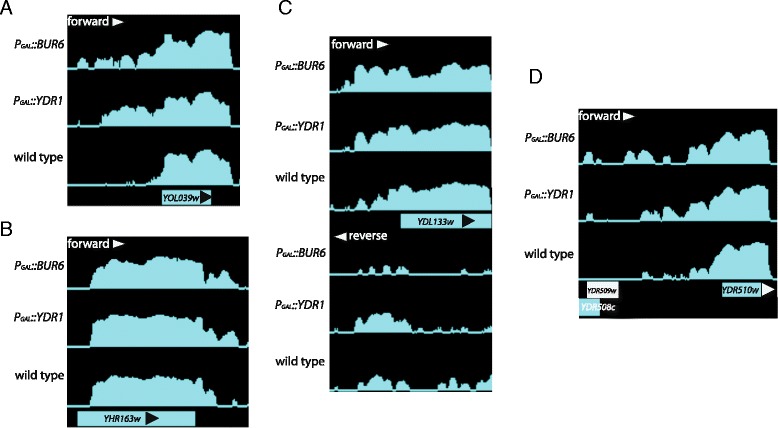
Selected examples of verified ORF-genes showing cryptic transcription in their promoters. Screenshots from the tiling analysis software (TAS) are shown as example of the different cryptic transcripts detected after shutting down either *BUR6* or *YDR1*. Intensity profiles on an arbitrary, but identical, scale for the mutants and wild type are shown. In (**a**, **b** and **d**), only the forward signal is shown, and both the forward and reverse intensity signals from the strand-specific microarray are shown in **c**. The SGD map for canonical genes is represented below and indicates the sense of transcription with an arrowhead. **a**) Cryptic transcription in the 5′ flank of the YOL039w/*RRP2A* gene in both NC2 mutants. **b**) Cryptic transcription in the 3′ flank of the YHR163w/*SOL3* gene in both NC2 mutants. **c**) Cryptic transcription in the 5′ flank of the YDL133w/*SRF1* gene in both NC2 mutants in both forward and reverse (antisense). **d**) Cryptic transcription in the 5′ flank of the YDR510w/*SMT3* gene in both NC2 mutants. In this case the cryptic transcript could be detected with a probe from the YDR509w dubious ORF as in the macroarray experiment described in Fig. 1 and in the text

**Fig. 4:**
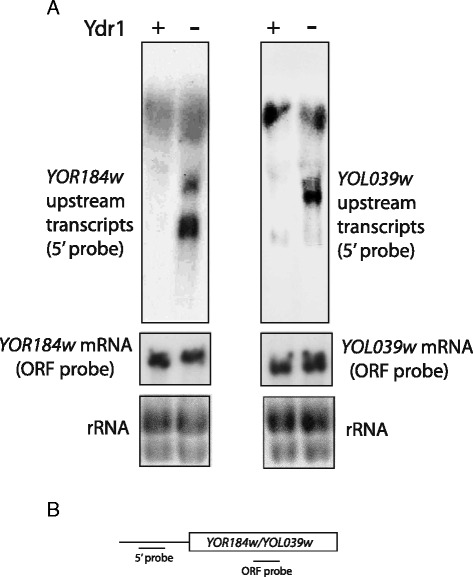
Short transcripts are detected upstream the YOR184W/*SER1* and YOL039W/*RPP2A* genes in conditions of Ydr1 depletion. Yeast strains FY86 (+Ydr1) and *P*_*GAL1*_-*YDR1* (−Ydr1) were grown in YPGal in the early exponential phase and then transferred to YPD and incubated for 4 h. **a**) Total RNA was prepared and analyzed by Northern blot using probes corresponding to the ORF or the 5′ upstream region. Ethidium bromide staining of ribosomal RNA (rRNA) was used as a loading control for the total amount of RNA. **b**) A scheme of the probes used is shown

In order to further support and validate our genome-wide conclusions, we visually inspected the tiling array data at the individual gene level to find particular genes with changes in expression in their promoter regions. Figure 3 shows four examples of canonical genes with a “dubious” ORF, or not, in their close proximity, which display a clear increase in expression in the comparison between NC2 mutants and the wild type. Moreover, in order to experimentally confirm that the lack of NC2 provokes cryptic transcription, we selected two cases in which the macroarray experiment found an increase in the upstream transcription: YOR184W/*SER1* and YOL039w/*RPP2A* (Fig. 4). The case of *SER1* is especially interesting because another gene involved in the serine + glycine biosynthesis pathway, *SER3*, has been shown to be regulated by a non-coding upstream transcript, *SRG1* [27]. As seen in Fig. 4, the shutoff of *YDR1* resulted in the appearance of small transcripts in the promoter region of both *SER1* and *RRP2A*.

## Discussion

RNA pol II transcribed genes, protein-coding as well as non-coding, may contain TATA or TATA-like elements in their promoters [3]. Both kind of promoters require TBP for PIC formation, although the mechanism for this purpose in TATA-like promoters is not yet clear [19].

NC2 and Mot1 are factors that seem to facilitate the interchange of TBP from DNA-bound to a free state, and vice versa [18, 28]. This kinetic effect on the TBP equilibrium between PIC and free states seems to facilitate the binding of TBP to the non-preferred sites of TATA-like promoters [18]. Thus Mot 1 and NC2 act as activators of TATA-like genes and repressors of TATA-containing genes. Recently, M. Timmers’ group has reported that depletion of the NC2 complex, in conjunction with mutations in chromatin remodeling or nucleosome deposition genes increases cryptic intragenic transcription [21]. This suggests that the cryptic TATA promoters that are blocked in a wild-type chromatin context are exposed in these mutants, and are used for PIC formation upon NC2 or Mot1 depletion. These authors also found, in the wild-type chromatin structure, that the simultaneous depletion of Mot1 or NC2 with the Nrd1-Nab3-Sen1 (NNS) complex involved in cryptic transcripts degradation increases the antisense transcript production that arises from the 3′ end of selected loci [22]. This finding suggests that the cryptic TATA promoters present at the 3′ end of most protein-coding genes in 3′-flank nucleosome-depleted regions are activated when TBP-interchange factors are absent.

Our study extends these observations to the genome-wide level by showing that the activation of cryptic transcription, in both the sense and antisense directions upon NC2 depletion, is a general phenomenon across the yeast genome. Ydr1 and Bur6, the two subunits of NC2, performed similar, but not identical, activities. The depletion of either subunit resulted in transcript accumulation at both ends of genes in a genome-wide average (Fig. 2). These peaks reflect the appearance of the short cryptic transcripts that initiated in the 5′ and 3′ nucleosome-free regions (NFRs) spanning regions of less than 200 nucleotides (see the model in Fig. 2i). Apart from these types of promoter- and terminator-associated transcripts in Fig. 2a & b, we also detect increased antisense transcription along the gene body. Depletion of Bur6 has a stronger effect on intragenic antisense transcription (Fig. 2b), whereas depletion of Ydr1 had a more marked effect on sense transcription in TATA-containing genes (Fig. 2c). This latter result presumably came from the relative activation of TATA-dependent genes under NC2 depletion conditions. In fact, we have observed that TATA genes are up-regulated upon Ydr1 depletion, whereas TATA-like genes do not change (1.06 factor). In contrast, no differences between TATA and TATA-like genes are found under the Bur6 depletion conditions (Fig. 2d). Thus it seems that the TBP-interchange function of NC2 in canonical TATA promoters depends more on Ydr1, but Bur6 is more important for the same function in cryptic (presumably TATA) promoters. It is noteworthy that we found no significant antisense transcription accumulations at the 3′ NFR of all the analyzed genes (protein-coding and non-coding). The observation of Koster and Timmers [22] that NC2 depletion increases antisense transcription from 3′ ends, and our NC2 depletion experiments showing the appearance of antisense transcription that covered the entire gene (Fig 2a & b, gray lines), without a localized accumulation at the 3′ end, support the existence of well-defined TATA promoters in the 3′ NFR regions that direct antisense transcription which overlaps the entire gene. Existence of cryptic promoters in 3′ NFRs has been demonstrated by different groups ([29]; discussed in [30]). These promoters direct the synthesis of the antisense Rco1-restricted transcripts (RRTs) identified by [31]. RRTs are suppressed by the action of Rpd3 histone deacetylase (HDAC) to avoid (at least partially) the antisense transcription caused by gene looping [30]. The cryptic transcripts at the 5′ end, associated with the TSS (SRT, Ssu72-restricted transcripts) are, however, independent of Rpd3 HDAC and dependent on Set3 HDAC [32, 33]. We hypothesize that presence of antisense peaks in 5′ regions in all analyzed cases (Fig. 2a, b, e–h) may be originated from the 3′ end of contiguous canonical genes and represent the extension of the RRT transcripts that started at the TATA sequences located at their terminator regions. These sequences are extremely abundant at the positions around 25–60 nucleotides upstream of the polyA site [34], direct the transcription inside the coding region, and would become activated in the NC2 depletion mutants (see Fig. 2i).

In our study we also analyzed ncRNAs, such as CUTs and SUTs (Fig. 2e–h). The average transcription level for these genes is much lower than for canonical genes, which results in noisier, less detailed metagene profiles. Moreover, the lack of a classification into TATA and TATA-like promoters for these transcripts prevents us from looking for promoter type-dependent differences. However, given these considerations, our results indicate that these genes are up-regulated in NC2 depletion mutants and also support the existence of flanking transcripts similar to those observed in canonical protein-coding genes.

## Conclusions

Given the known function of NC2 in the TBP interchange from free-to promoter-bound states, and also the known role of the TBP protein in determining TSS recognition, it is reasonable to think that NC2 plays an important role during precise start site selection. Our results suggest that NC2 plays a very general role in TSS selection for RNA pol II in all kind of genes it transcribes. The ablation of the NC2 function increases cryptic transcription genome-wide, likely as a result of TBP being able to bind to any accessible TATA promoter with greater stability than in the presence of an active NC2 complex. Thus we hypothesize that the cryptic TATA promoters which are infrequently used in wild-type cells become more active in our shutoff mutants. Access to these kinds of promoters is much easier within the NFRs located at the 5′ and 3′ flanks of RNA pol II genes [26, 29]. The cryptic transcripts that arise from them are probably then substrates for fast degradation by the nuclear quality control pathway [35]. Based on our results, we have further evidence to suggest functional differences between the two NC2 subunits; Ydr1 seems to be more important to TSS determination in protein-coding genes, and Bur6 in non-coding transcripts. It has been shown previously that NC2 subunits play different roles in transcription [36]. In fact, those authors showed that subunits Ydr1 and Bur6 are not associated in a stable complex in exponentially growing cells, and are differentially present at gene promoters. Our results are an independent proof of the existence of various NC2-TBP complexes in vivo. Nonetheless, future studies will continue to extend our knowledge about the particular roles of NC2 subunits during transcription initiation.

## Methods

### Yeast strains, DNA recombinant work, and microbiological techniques

Yeast strain FY86 (*MAT*α, *ura3*-*52*, *leu2*Δ*1*, *his3*Δ*200*; Winston et al., [37]) was used to construct strains *P*_*GAL10*_-*BUR6* and *P*_*GAL10*_-*YDR1* by respectively replacing the wild-type promoter of genes *BUR6* and *YDR1* with the *GAL10* promoter, including three copies of the hemagglutinin (HA) epitope [23] as previously described in [24].

Yeast cells were grown overnight at 28 °C in YPGal (Yeast extract 1 %, Peptone 2 %, Galactose 2 %) in the early exponential phase (OD_600_ between 0.2-0.5). At that time cells were recovered by centrifugation and resuspended in YPD (same medium, but 2 % Glucose instead of Galactose) and were grown for another 4-h period before RNA extraction.

### RNA extraction and Northern blot

Cells were harvested, washed with water, and frozen at−80 °C. RNA extraction and the Northern blot analysis have been previously described elsewhere [38].

### Macroarray gene expression analysis

*S. cerevisiae* DNA macrochips in nylon membranes [25] were used for the transcriptome analysis. Then 40 μg of total RNA were used to synthesize radiolabeled cDNA using 500 ng of oligo dT (T_15_/VN) as in [39]. Three replicates of each strain were used. Scanning and bioinformatics analyses were done as in [40].

### Tiling array hybridization and analysis

5 μg of total RNAs were labeled following the GeneChip® Whole Transcript (WT) Sense Target Labeling Assay Manual of Affymetrix to hybridize the Custom Tiling Array (PN 520055, Affymetrix, Santa Clara, CA, USA [39]). Three replicates of each strain were used and their results averaged.

Raw. CEL images were processed by the Tiling Analysis Software (TAS, Affymetrix) with the signal detection parameters set by default. To visually inspect the hybridization signals in relation to the annotations from the *S. cerevisiae* reference genomic map, the Integrated Genome Browser (http://bioviz.org/igb/index.html) software was used. Both the “TilingArray” Bioconductor (http://www.bioconductor.org/packages/2.11/bioc/html/tilingArray.html) and custom R scripting packages where used for the metagene analysis and scatterplot generation.

### Availability of supporting data

The data sets that support the results of this article are available in the GEO repository [http://www.ncbi.nlm.nih.gov/geo/] with accession numbers GSE17303 for the macroarray data and GSE67267 for the tiling array data.

## Abbreviations

CUT: Cryptic unstable transcripts
GTFs: General transcription factors
HDAC: Histone deacetylase complex
NC2: Negative cofactor 2
ncRNA: Non-coding RNA
NFR: Nucleosome-free region
NNS: Nrd1-Nab3-Sen1
ORF: Open reading frame
pA: Polyadenylation sites
PIC: Preinitiation complex
RNA pol II: RNA polymerase II
rRNA: ribosomal RNA
RRT: Rco1-restricted transcripts
SAGA: Spt-Ada-Gcn5-acetyltransferase
SRT: Ssu72-restricted transcripts
SUT: Stable untranslated transcripts
TAFs: TBP-associated factors
TBP: TATA-binding protein
TSS: Transcription start sites
